# Intratumor Heterogeneity: A Central Challenge in Modern Oncology

**DOI:** 10.3390/cancers18101531

**Published:** 2026-05-09

**Authors:** Constantin N. Baxevanis, Ourania E. Tsitsilonis, Angelos D. Gritzapis

**Affiliations:** 1 Cancer Immunology and Immunotherapy Center, St. Savas Cancer Hospital, 171 Alexandras Avenue, 11522 Athens, Greece; agkritzapis@agsavvas-hosp.gr; 2 Flow Cytometry Unit, Department of Biology, National and Kapodistrian University of Athens, Panepistimiopolis, Ilissia, 15784 Athens, Greece; rtsitsil@biol.uoa.gr

Cancer has traditionally been described as a disease driven by uncontrolled cell growth caused by the gradual accumulation of genetic alterations. For many years, tumors were viewed as relatively uniform masses originating from a single transformed cell. Although this model helped early discoveries in cancer biology, it is now clear that it fails to reflect the entire complexity of tumor development. With the scientific advances in high-throughput sequencing, single-cell technologies, and multi-omics approaches, a different picture has emerged. Tumors are not uniform cell populations; instead, they consist of diverse subclones with distinct genetic, molecular and functional characteristics. This diversity, referred to as intratumor heterogeneity, is now widely recognized as a fundamental feature of cancer, spanning from genetic mutations to epigenetic regulation, transcriptional states, as well as phenotypic and metabolic profiles [1]. Intratumor heterogeneity is widely recognized as a fundamental feature of cancer, encompassing genetic mutations, epigenetic regulation, transcriptional states, and phenotypic and metabolic diversity. It significantly influences tumor growth, evolution, and treatment response, representing a major barrier to effective therapies while also motivating personalized cancer treatment strategies [1,2].

Tumors are dynamic systems in which subclonal populations coexist and evolve under environmental and therapeutic selective pressures. Intratumor heterogeneity varies widely between patients, cancer types, and even within different regions of the same tumor, with mutational profiles that can change over time, particularly under treatment, often leading to the expansion of resistant subclones [3,4,5]. This variability complicates disease prediction and treatment decisions, highlighting the need for real-time monitoring to guide therapy. These observations suggest that tumor progression is not always a unidirectional process but can involve flexible and reversible transitions between distinct cell types [6,7]. Tumor heterogeneity arises from genomic instability, which generates mutations, and selection, which shapes clonal expansion, as described by the clonal evolution model [8]. However, genetic changes alone do not fully explain tumor diversity, as non-genetic mechanisms such as epigenetic and transcriptional variability also contribute by enabling phenotypic plasticity without altering DNA sequence. This plasticity allows cancer cells to adapt to stress and therapy, sometimes adopting hybrid states that enhance survival, invasion, and resistance, indicating that tumor progression can involve flexible and reversible cellular transitions [8,9].

The tumor microenvironment plays a key role in driving tumor heterogeneity, as cancer cells interact with stromal cells, immune cells, and extracellular matrix components in a dynamic ecosystem [10,11]. Local differences in oxygen, nutrients, and immune activity create selective pressures that favor different subclones, with hypoxia promoting metabolic adaptation and immune pressure selecting for less immunogenic variants [12]. Immune and stromal cells can further shape tumor behavior, contributing to cancer immunoediting and influencing whether tumor growth is supported or restricted depending on their functional states [13,14,15,16]. Recent advances such as single-cell sequencing, spatial transcriptomics, and advanced imaging have greatly improved the resolution at which tumor heterogeneity can be studied by revealing rare cell populations and preserving spatial context. When combined with computational and machine learning approaches, these technologies are enabling predictive models of tumor behavior and treatment response, supporting the development of more adaptive therapeutic strategies [17,18,19]. From a clinical perspective, intratumor heterogeneity creates major challenges, particularly treatment failure. Therapies targeting a single pathway often fail to eradicate all tumor cell populations, allowing less responsive clones to persist and drive relapse across targeted therapy, chemotherapy, and immunotherapy [20,21,22]. Heterogeneity also complicates diagnosis and biomarker discovery, since standard biopsies sample only a limited tumor region and may miss subclones linked to treatment insensitivity, metastasis, or aggressive behavior. As a result, biomarkers may not reflect the full tumor landscape, leading to suboptimal treatment decisions and poorer outcomes. To address these limitations, new approaches such as analysis of circulating tumor cells and DNA and single-cell RNA sequencing enable more comprehensive, real-time monitoring of tumor evolution [23]. Advances in imaging and radiomics further improve the assessment of spatial heterogeneity across the entire tumor [24,25]. Improved knowledge of intratumor heterogeneity has stimulated the development of novel treatment strategies. Combination therapies that target multiple pathways simultaneously may reduce the development of resistance [26,27]. In addition, adaptive therapies can optimize treatment, based on how a patient’s tumor evolves, aiming to suppress the growth of resistant tumor subclones and extend treatment effectiveness, ultimately improving clinical efficacy [28,29]. Both strategies provide new platforms toward more flexible therapeutic modalities which take tumor evolution into account. To this end, integrating real-time monitoring with predictive algorithms may allow treatments to be adjusted accordingly as tumors evolve. Data from liquid biopsies and imaging could help guide these decisions, allowing changes within the tumor to be more responsive to therapy [30]. While many challenges remain, including the need for reliable and clinically validated models, this direction holds considerable promise.

## Future Perspectives: From Heterogeneity to Therapeutic Opportunity

Looking forward, intratumor heterogeneity should be reframed not only as a barrier to successful cancer treatment but also as a potentially exploitable source of therapeutic opportunity. The same diversity that enables tumor cells to adapt and evade therapy also creates inherent vulnerabilities that may be targeted if properly understood and monitored over time. Rather than viewing heterogeneity solely as a complication, it can instead be considered a dynamic feature of cancer biology that offers multiple intervention points throughout disease progression. Several future directions are therefore particularly promising for translating this concept into clinical benefit. First, real-time molecular monitoring is likely to become a standard component of oncology practice. Continuous or repeated profiling using circulating tumor DNA and circulating tumor cells, together with advanced imaging-based biomarkers, could enable clinicians to track tumor evolution as it occurs. This would allow treatment strategies to be dynamically adjusted in response to emerging changes in tumor composition, rather than relying on fixed and static therapeutic protocols that may quickly become outdated as the tumor evolves. Second, the integration of artificial intelligence, computational approaches, and mechanistic evolutionary modeling will be essential for interpreting the increasingly complex and multidimensional datasets generated by modern cancer profiling technologies. Machine learning approaches, when combined with principles of clonal evolution and tumor ecology, may allow the prediction of resistance pathways before they fully emerge, potentially enabling preemptive therapeutic interventions that delay or prevent treatment failure. Third, the development of adaptive clinical trial designs will be required to properly evaluate therapies in a context that reflects the dynamic nature of tumor evolution rather than traditional static endpoints. Such designs may include flexible dosing strategies, response-guided treatment modifications, and longitudinal sampling protocols that capture tumor changes over time. Together, these approaches represent a shift toward more personalized, evolution-aware oncology strategies that aim not only to treat cancer but also to anticipate and manage its continuous adaptation.

## Conclusions

Intratumor heterogeneity should not be solely considered as an obstacle to overcome, but also as a way to reconsider how cancer cells could be effectively targeted. The genetically and functionally diverse cell populations that make up a tumor interact with one another and with their surroundings, both the immune and non-immune microenvironment. Gradually, they shift and adapt in response to internal as well as external pressures, also including therapeutic treatment. Recognizing this complexity helps explain why treatments can lose their effectiveness with time and why resistance often develops. Taking tumor evolution into account paves the way for designing treatment strategies that are more adaptable. Instead of using a single therapeutic treatment, approaches like combination therapies and personalized or sequenced treatment strategies can help control tumor growth rates over time. The aim is not only to reduce tumor burden but also to suppress mechanistic pathways that allow resistant subclones to develop. To achieve this, interdisciplinary contributions are required. For instance, translational research is essential for uncovering and exploring the mechanisms that drive diversity within tumors. Equally important, computational methods can help analyze complex data and reveal how tumors change over time. Finally, clinical application remains central for assessing how well different strategies perform in patients. Integrating these perspectives will be essential to move the field forward. Addressing intratumor heterogeneity is closely linked to reducing relapses, managing the onset of resistance, and achieving long lasting clinical outcomes. As knowledge in this field grows, it is becoming more evident that effective cancer therapy depends not only on targeting the tumor itself, but also understanding and responding to the internal diversity of the tumor over time.
